# Rice husk reuse as a sustainable energy alternative in Tolima, Colombia

**DOI:** 10.1038/s41598-024-60115-5

**Published:** 2024-05-06

**Authors:** Angie Tatiana Ortega Ramírez, Miriam Reyes Tovar, Oscar Silva-Marrufo

**Affiliations:** 1 Faculty of Engineering, Universidad de América, Bogotá, DC Colombia; 2 PhD Sustainability, Universidad Centro Panamericano de Estudios Superiores (UNICEPES), Michoacán, Mexico; 3 Faculty of Cultural, Demographic and Political Studies, Universidad de Guanajuato, Guanajuato, Mexico; 4 Faculty of Engineering, Tecnológico del Valle del Guadiana, Durango, Mexico

**Keywords:** Rice husk, Bioenergy, Mixed methodology, System Advisor Model (SAM), Combustion, Bioproducts, Energy efficiency, Tolima, Environmental sciences, Engineering

## Abstract

Colombia has great potential to produce clean energy through the use of residual biomass from the agricultural sector, such as residues obtained from the life cycle of rice production. This document presents a mixed approach methodology study to examine the combustion of rice husks as a possible energy alternative in the Tolima department of Colombia. First, the physicochemical characteristics of the rice husk were analyzed to characterize the raw material. Next, System Advisor Model (SAM) software was used to model a bioenergy plant to obtain biochar, bio-oil, and biogas from the combustion of rice husks and generate performance matrices, such as thermal efficiency, heat rate, and capacity factor. Then, the project was evaluated for financial feasibility using a mathematical model of net present value (NPV) with a planning horizon of 5 years. Finally, a subset of the local population was surveyed to assess perspectives on the project in the region. The results of the rice husk physicochemical analysis were the following: nitrogen content (0.74%), organic carbon (38.04%), silica (18.39%), humidity determination (7.68%), ash (19.4%), presence of carbonates (< 0.01%), and pH (6.41). These properties are adequate for the combustion process. The SAM simulation showed that the heat transferred in the boiler was 3180 kW, maintaining an efficiency between 50 and 52% throughout the 12 months of the year, meaning that the rice husk can generate electricity and thermal energy. The financial analysis showed that the internal rate of return (IRR) was 6% higher than the opportunity interest rate (OIR), demonstrating economic feasibility of the project. The design and creation of a rice husk processing plant is socially and environmentally viable and has the potential to contribute to the economic development of the Tolima community and reduce greenhouse gases. Likewise, this activity has the potential to promote energy security for consumers and environmental sustainability while at the same time being economically competitive.

## Introduction

**Figure 1:**
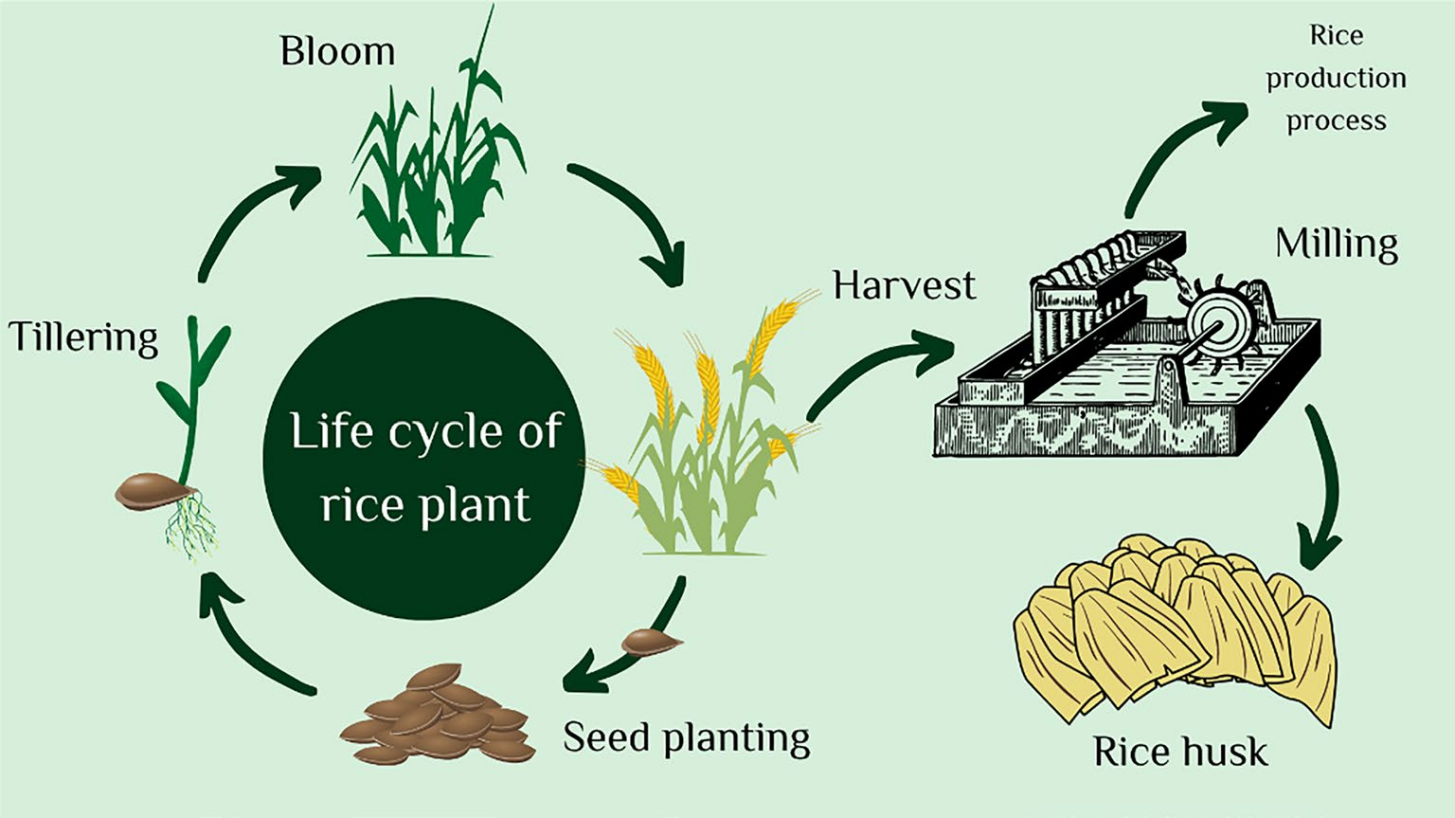
Rice life cycle, emphasizing rice husk residue.

Rice cultivation began approximately 10,000 years ago in various regions of Asia and has become the second largest crop worldwide after wheat^1^. Between 2013 and 2019, Colombia ranked as the 22nd highest rice-producing country in the world, with an average production of 2.71 million tons according to data from the Food and Agriculture Organization of the United Nations (FAO)^2^. In 2019, Colombia had a rice harvest area of 518,000 ha, which increased by 12% in 2020, reaching 580,000 ha. Similarly, the market share increased by 40% in 2020 compared to 2019. This growth corresponded to an increase in productivity and yield in all rice-growing areas of Colombia and an increase in producer prices^3^. Rice production involves several steps, the main ones of which are harvesting, drying, and husking. Husking is the step that generates the greatest amount of waste^4^. The life cycle of rice begins with the sowing of grain seeds, then the growth of its shoots and subsequent flowering, the harvesting of the grains, and finally, the start of another cycle as shown in Fig. 1.

The department of Tolima, whose capital is Ibagué, is in the center of the country at coordinates 4° 26′ 00″ N 75° 14′ 00″ W^5^. The department, with an area of 23,562 km^2^, has 47 municipalities and borders the departments of Caldas, Quindío, Risaralda, and Valle del Cauca. The department is traversed by the Magdalena River and is home to other water basins such as: Gualí, Sabandija, Recio, Lagunillas, Opía, Anchique, Chenche, and Atá. This area is characterized by a semi-humid climate due to its varying annual rainfall^6^. Due to the great diversity of soils and climates, Tolima is considered to have significant natural wealth, with various crops harvested here along with a growing industrial development^7^. In Tolima, rice production reached 406,731 tons, which is reflected in a growth of 12.2% compared to the second half of 2019^8^.

In Tolima, the National Administrative Department of Statistics reported an annual rice production of 726,785 tons for 2019^9^. In this year, the department produced 24.4% of the total rice production in the country; however, the same report identified a decrease of 9.5% in rice production in Tolima with respect to 2018. This is perhaps due to a decrease in the area planted, which for 2019 was 97,155 ha, with a decrease of 8.7% with respect to 2018. However, the 2019 rice crop yield increased by 0.8% with respect to 2018, with 7.5 tons of rice produced per hectare^10^ somewhat offsetting the decrease in harvested area.

In Colombia, an estimated 6,282,407 tons of rice residues are generated per year^11^, mainly generated chaff and rice husk. The husk residue is approximately 20% of the total rice production. It does not easily decompose and so, for each kg of rice grain recovered, 1.5 kg of rice chaff is produced^11^.

In Colombia, rice plant chaff currently does not have many applications, unlike rice husk, which has various uses. Less than 5% of this residue is used in stables, poultry farming, gardening, and other agricultural activities^11,12^.

However, the chemical and mineralogical composition of rice husk residues has traditionally not been considered. These properties, in addition to the usable energy power of this residue, potentially make these residues apt for biofuel generation, which would enable a more sustainable management of rice husk residues, based on studies previously elaborated and validated by various laboratory tests performed during this study. Using risk husk residues for biofuel generation would also promote the circular economy instead of continuing with the traditional linear economy, in which these biomass residues are usually disposed of in the sea or incinerated without seeking sustainable alternatives. Rice husks generate 492,738 tons of waste per year, which represents 7,136.53 Tera Joules of energy per year that are not being used to their maximum potential due to lack of information on non-conventional renewable energy sources.

Life Cycle Analysis (LCA) is a tool specifically designed to evaluate the environmental impacts related to the production chain of a good and make recommendations on how to improve the process in search of a more sustainable approach. During this study, this analytical tool was used to evaluate by-products of the rice industry, such as rice husks^13^. A grain of rice is mainly composed of the endosperm and the embryo. During the growth of rice, the husks form as a coating or protective layer of the grain^14^. The main solid residues that are generated during rice production are straw, husk, and ash. The rice husk is a plant tissue made up of cellulose and silica, elements that contribute to its good performance as fuel. The silica composition of rice husk is approximately 72%, which can increase when this biomass is burned, obtaining values of up to 95%^15^. The husk presents a variety of physicochemical characteristics, which are studied according to the application to be given. The husk is usually considered waste or agro-industrial residue because it is the part that surrounds the rice grain and generates inconveniences for rice producers. However, rice husk currently has different uses, such as bedding in poultry houses, mangers, flowers, concentrated food, composting, and production of biofuels. In addition to this, it is used to control excess moisture when preparing fermented fertilizers. Rice husk briquettes can also be made with rice husks, which minimizes costs in energy production. Kaolinized rice husk can also be used, improving moisture retention in hydroponic crops^16^.

The most important by-product or rice is the husk, which represents about 20% of the grain^17^. The proper use and disposal of rice husks represents one of the main challenges facing the Colombian rice industry. Currently, the husks are sold to third parties at low prices as livestock feed^18^. However, given the new energy transition policy^19^, and with an aim to mitigate environmental impacts of the industry, this project was developed to evaluate the transformation and use of biomass from rice husks using a pyrolysis process and thus identify their energy potential and use as a sustainable energy alternative in Tolima, Colombia.

The current predominant forms of energy are produced using a variety of fossil fuels, which contribute to global warming through production of greenhouse gases^20^. Recently, there has been an emphasis on clean energy production, which contributes to a reduction in the carbon footprint.

Colombia has potential to produce energy through residual biomass from the agricultural sector, such as residues from the production of sugar cane, oil palm, coffee, corn, bananas, and rice^21^. This study analyzed the production of electrical energy through rice husk pyrolysis while considering the local Colombian context and analyses related to the life cycle of rice production, specifically in the recovery of residues such as rice husks, linking it to circular economy strategies. The study proposed an energy sustainability strategy to link activities that promote the triple account of sustainability, which has become increasingly relevant in various Latin American countries including Colombia^22^. The purpose of the strategy is to promote non-conventional renewable energies so they can enter the energy market. The renewable energy sector has increased in recent years, reaching up to 6% of world investments, which helps reduce carbon emissions^23^.

The applicability of an LCA in the agri-food sector highlights environmental hotspots, specifically those activities or processes responsible for environmental impacts^24^. In addition, the LCA can be applied to calculate an energy balance and recognize the main categories of environmental impact in an agronomic process, such as climate change, waste management, and soil acidification. Similarly, an LCA can provide analysis and identification of better technologies in agricultural systems, such as the pyrolysis of rice husks to produce energy, and their economic, social, and environmental benefits^25^.

## Methods

**Figure 2:**
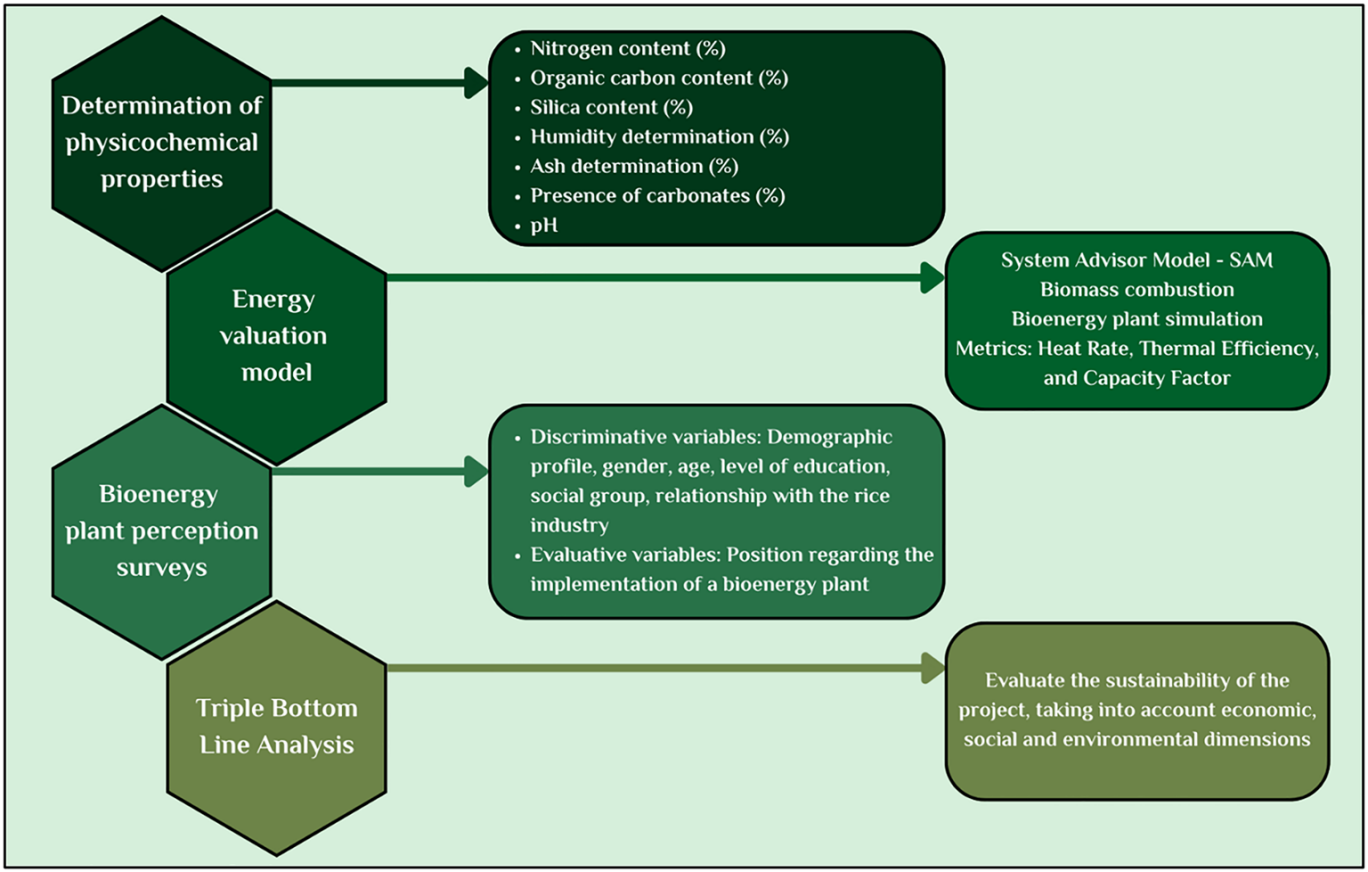
Mixed methodology with both qualitative and quantitative approaches.

The study was implemented through mixed research methodologies as shown in Fig. 2, with both qualitative approaches represented by interviews conducted with members of the local population and representatives from the rice companies and quantitative approaches represented by the gathering and analysis of data for the SAM simulator to design a clean energy production plant. The rice husks were supplied by the farmers in the area. The mixed methodology was divided into four phases, described below:

### Determination of physicochemical properties

The collected rice husk samples were analyzed to determine the following physicochemical properties: nitrogen content (%), organic carbon (%), silica (%), moisture determination (%), ash (%), presence of carbonates (%), and pH. Table 1 specifies the method or analytical technique used for each property. The laboratory tests were performed by Chemilab, a laboratory accredited by the Institute of Hydrology, Meteorology and Environmental Studies (IDEAM).

**Table 1:** Physicochemical parameters of rice husk.

Parameter	Result	Method—analytical technique
Total nitrogen	0.74%	Semi-Micro Kjeldahl
Organic carbon	38.04%	IGAG
Silica	18.39%	SM 4500 Si D
Carbonates	< 0.01%	IGAG – Potentiometric titration
Ash	19.4%	Calcination at 550 °C
Natural humidity	7.68%	ASTM D2216-98—Gravimetric
pH	6.41	NTC 5264—Electrometry

### Rice husk processing plant technical proposal

**Figure 3:**
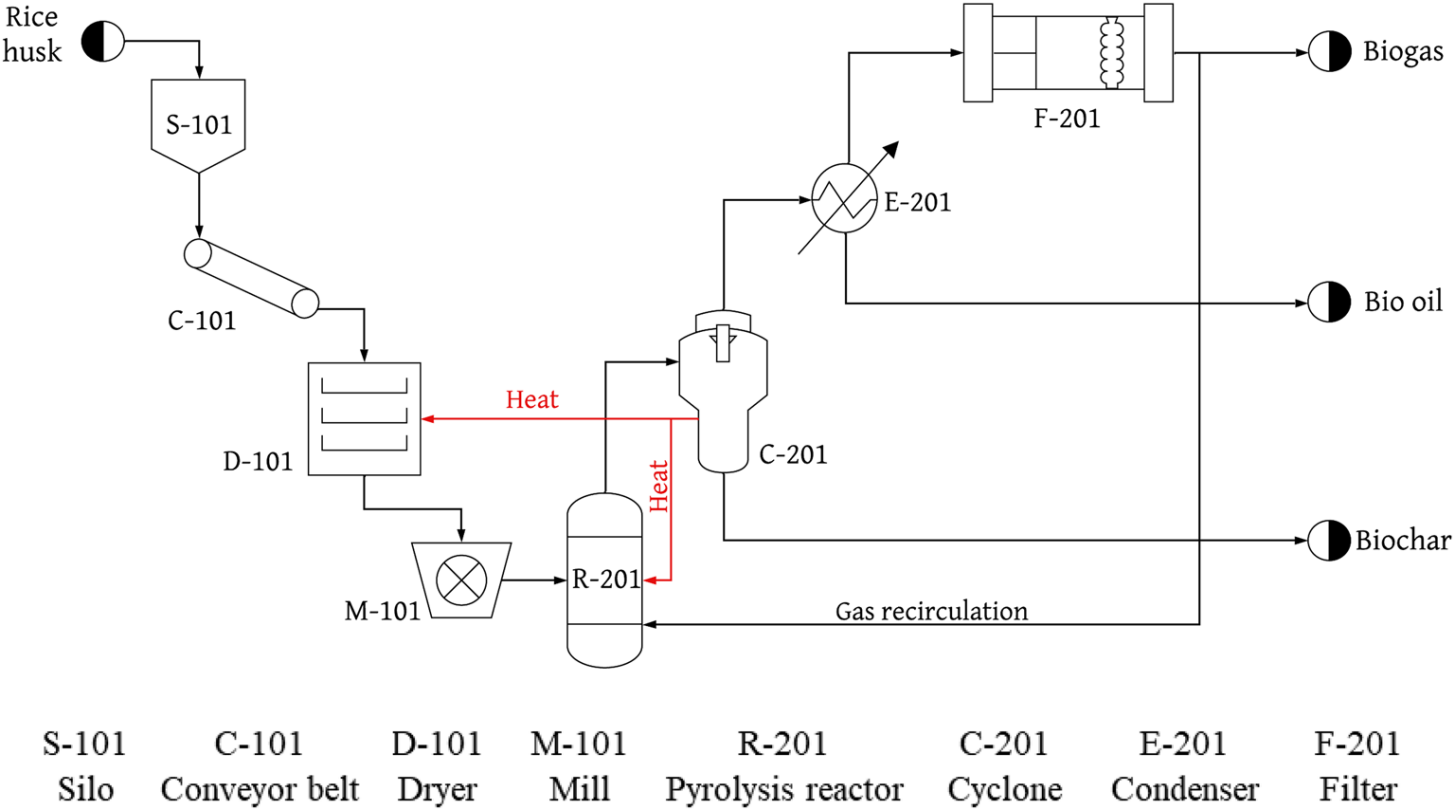
Process flow diagram (PFD) of the rice husk pyrolysis process.

The chemical plant would adopt a pyrolysis process, shown in Fig. 3, to transform biomass into bio-oil, biogas, and biochar. The plant would be developed through private investment and will be located in the municipality of Espinal in Tolima. The project is predicted to be accepted locally due to its focus on biofuels of renewable origin, which offer greater energy security, lower pollution emissions, and promote the development of the Colombian countryside.

The plant would have proper pre-treatment of the raw material, which would begin with the collection and storage of the rice husks in silos (S-101) at room temperature. The husks would then be transported by conveyor belt (C-101) to a dryer (D-101) at 100 °C^26^. Later, they would go to a hammer mill (M-101) at which point the rice husks would be ready to be transported to the pyrolysis reactor (R-201) at a temperature of 400 °C^27^. In order to separate the husks, it is necessary to pass the gas stream through a cyclone (C-201) (biochar), then a condenser (E-201) (bio-oil), and finally through a gas filter (F-201) (biogas). Using a systems approach, the recirculation of heavy gases to the pyrolysis reactor (R-201) would be implemented as well as the use of the heat dissipated in the cyclone (C-201) to be used in the drying process (D-101) and to make a re-entry into the reactor (R-201). The rice husk processing plant would enable the creation of economically and environmentally sustainable clean energy, giving value to the residues of the rice industry, guaranteeing the use of rice husks, and minimizing waste. On a social level, the project would provide development and employment opportunities, increasing the human capital of the region by supporting entrepreneurship projects and social, cultural, recreational, and sports programs.

However, for the operation of the production plant, the availability of the material should be evaluated since it is necessary to guarantee a large amount of rice husk, which will depend on agricultural production and harvest times. In the same way, the infrastructure represented by the construction of the plant must be considered, due to the population growth in the area and the increase in energy demand in the region.

### Energy valuation model

During this phase, the System Advisor Model (SAM) simulator was used to validate and simulate collected information. The SAM simulator is a techno-economic software designed by the National Renewable Energy Laboratory that facilitates decision-making in renewable energy projects^28^.

The SAM simulator presents a biomass combustion component that allows for the modeling of bioenergy plants that use crop residues and wood as raw material, facilitating the modeling of biomass combustion energy systems and generating performance metrics such as heat rate, thermal efficiency, and factor of capacity. Additionally, the SAM simulator can evaluate the financial viability of a project through financial indicators such as the leveling cost of energy (LCOE), the net present value (NPV), and the collection period^29^. The SAM simulator was used in this project to analyze an energetic model of the rice husk by considering the physicochemical properties obtained by previously described laboratory tests. The following inputs were needed for the analysis: location, system design parameters, and biomass properties through which the estimated capacity of a biomass power plant was determined, as well as capacity factor, rate of heat, and power produced^30^.

The internal calculations in the SAM bioenergy model use a different time scale for each calculated parameter. Therefore, moisture content is calculated by months whereas ambient temperature is calculated by hours and then averaged monthly since the efficiency of the boiler and the turbines varies with respect to this variable^31^.

This project focused on the concept of circular economy where inputs, waste, emissions, and energy losses are minimized by slowing down, closing, and reducing material and energy cycles^10^, achieving a change in global economic paradigms dominated by a linear model of production and consumption, in which products are manufactured by using raw materials that are then sold, used, and finally discarded as waste^32^. The circular economy aims to ensure that products, components, and materials maintain their maximum usefulness and value at all times by applying the 3R rule—Reduce, Reuse, and Recycle.

The economic system surrounding rice cultivation and the background of the project was reviewed in detail, evaluating the potential of rice husks as raw material for another productive process and/or energy generator. For this, the rice husks were characterized through a series of laboratory tests performed on a sample of rice husks obtained from a rice crop from Tolima.

Subsequently, an energy model of the rice husk was analyzed taking into account the physicochemical properties obtained as a result of the laboratory tests described above; this analysis was performed using the SAM simulator which required as input data the location, system design parameters, and biomass properties through which it determined the estimated capacity of a biomass power plant, as well as its capacity factor, heat rate, and energy produced.

The internal calculations in the SAM bioenergy model use a different time scale in each calculated parameter; thus, the moisture content is calculated on a monthly time scale, the ambient temperature is calculated by hours and averaged monthly because the efficiency of the boiler and turbines varies with respect to this variable. The following is a description of the basic internal calculations that underlie the analysis performed by the biomass energy module of the SAM simulator, as described in its technical manual^31^.

### Bioenergy plant perception surveys

Given the importance of social aspects for this project, the communities surrounding the rice zone of Tolima were surveyed with the objective of identifying the degree of connection of the residents with the rice industry, farming, knowledge about local agricultural activity, and finally, degree of interest in the development of a rice husk processing plant^33^**.**

A survey was conducted aimed at various social groups present in the study area that have a direct or indirect relationship with the rice industry in Tolima. The survey presented two main types of variables: discriminant and evaluative. Discriminant variables have the objective of demographically profiling people by gender, age, academic level, relationship with the rice-producing industry, and social group. Evaluative variables serve to record the assessment of those surveyed on the implementation of a rice husk transformation plant in search of a sustainable energy alternative^34^.

To perform correct statistical analysis of the information collected through the survey, a data analysis methodology was used which sought to integrate statistical techniques such as measures of central tendency and dispersion, and computer science for data analysis.

The SAM simulation showed that the heat transferred inside the engine of the boiler where the rice husk combustion occurred was constant over 3180 kW, while the efficiency of the reboiler oscillated between 50 and 52%, both as a function of time, which are the 12 months of the year. This finding helped to fulfill the third objective of this research, which was to establish the energy valorization of the rice husk by means of different simulations of a combustion process. In January, February, April, May, June, July, August, September, and October, the maximum heat values were reached near the middle of the month with magnitudes of 3230–3240 kW. In March, November, and December, the curve was less pronounced, implying that the heat released was not the highest at that time, at approximately 3220 kW. Based on the simulation, it should be emphasized that the operation of the engine was efficient over time, since the heat released was similar from one day to another and also from one month to another and, therefore, allowed the combustion process to occur optimally regardless of the time in which this equipment was being implemented, since it did not lose functionality. Therefore, this equipment could be used in a rice husk processing plant since it meets the energy requirements of the process.

### Triple bottom line (TPL)

The Triple Bottom Line (TPL) was a methodology designed by John Elkington in 1998 to assess sustainability and measure performance beyond traditional measures of profit, return on investment, and shareholder value, so that environmental and social dimensions are included^35^. The idea of the TPL is to satisfy the demands of the stakeholders in a project by understanding the economic, social, and environmental results. The sustainability of the project is evaluated by measuring impact, with emphasis on environmental capillary, social capital, profitability, and social responsibility of the various interest groups involved in the project^36^:

- Economic factor: The economic variables evaluated in the project are related to technical-financial aspects such as project financing, operating and non-operating expenses, and local economic development.
- Social factor: During the development of a project, it is important to evaluate the impact on local communities, such as, in the case of this project, Colombian regulations for agricultural activities. These interests may influence the social acceptance of the project.
- Environmental factor: Environmental variables consist of those characteristics that can be evaluated to promote a healthy environment and energy security in the area.

It is necessary to take into account the social component in this research through the perception of the inhabitants in order to analyze the three approaches to sustainability (social, economic, and environmental).

## Results

After applying the methodology explained in the previous section, the next step was to describe the results obtained and perform a Life Cycle Analysis with an approach directed to the analysis of the TPL.

### Physicochemical properties of rice husk from Tolima, Colombia

Table 1 presents the results of the analysis performed by the Chemilab laboratory to measure certain physicochemical properties of the rice husk from Tolima. Knowing these properties allowed for the measurement of the energy potential of rice husk as biomass for the creation of biofuel. The Tolima rice husks had a high silica content, negatively affecting the energy value. Pretreatment to remove silica is, in general, a good practice when producing energy from biomass^37^. In the next section, these data were used to run the SAM simulator.

### Energetic behavior in the combustion process

**Figure 4:**
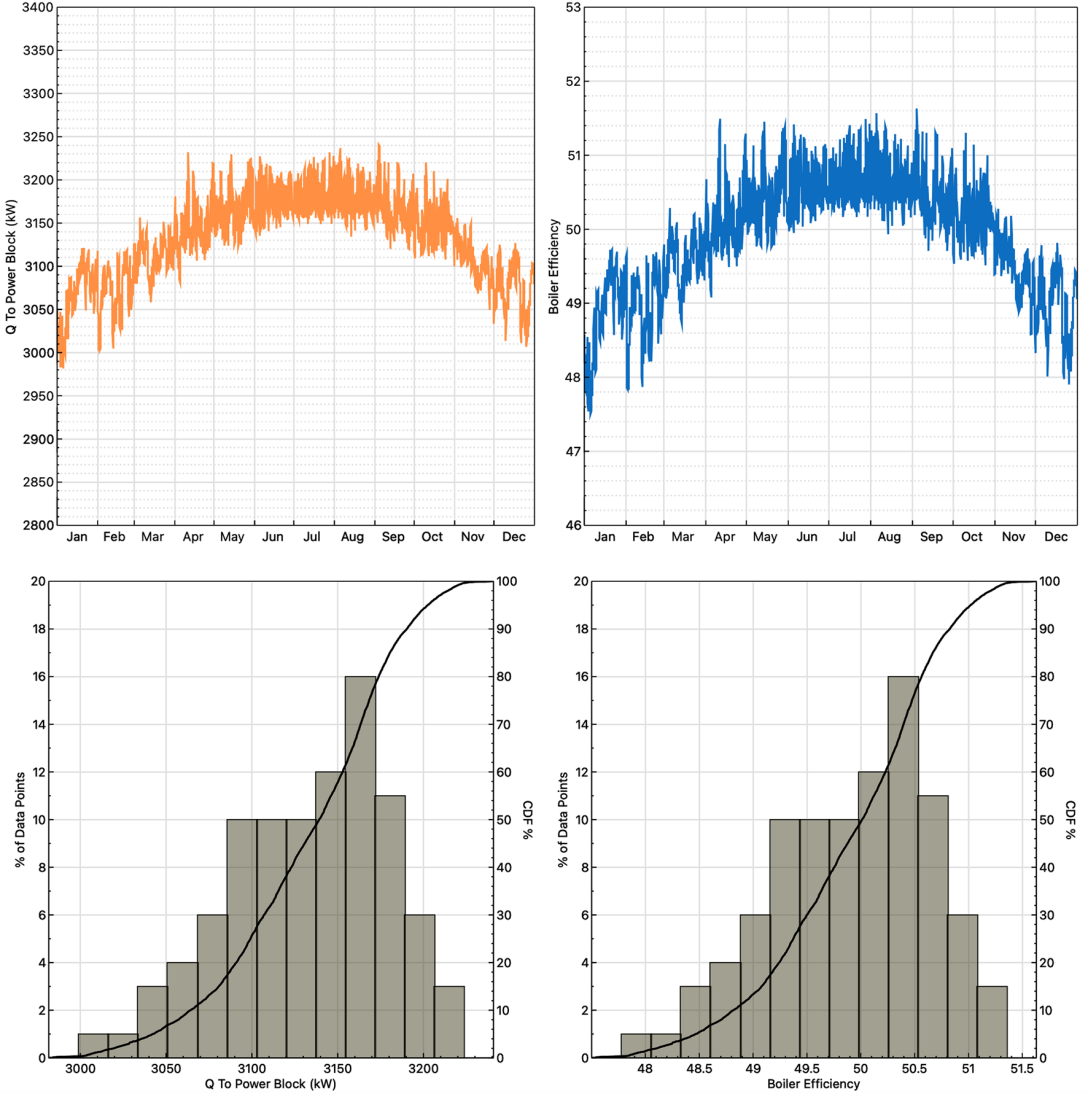
Graphs of the combustion process of rice husk over 12 months in the SAM simulator.

The energetic behavior of the rice husk in the combustion process was evaluated using the SAM simulator, which showed that the heat transferred inside the boiler engine where combustion of the rice husk occurred was constant at over 3180 kW, while the efficiency of the reboiler oscillated between 50 and 52 percent. Both variables were dependent on time and varied throughout the year, as can be seen in Fig. 4.

The results show that the operation of the engine can be effective over time, since the heat released was similar from one day to the next and also from one month to the next and, therefore, allowed the process of combustion to be performed optimally regardless of the time in which said equipment was being implemented by not losing functionality. Observing constant results over time indicates that the operation of the rice husk processing plant to produce clean energy could be efficient and constant over time and would not have great variability in the results.

### Demographic validation

For this research, stakeholders directly or indirectly related to the rice industry in Tolima were surveyed to understand their impressions and opinions of implementing a rice husk processing plant. The number of participants were 30, of which 10 were direct workers (33.33%), 10 rice farmers (33.33%), and 10 civilians (33.33%). The population sample consisted of 56.70% men and 43.40% women. The participants indicted knowledge of the use of rice husk for various purposes, such as the fabrication of fertilizers (60.00%), biomass (23.30%), construction material (6.70%), and fuel (10.00%).

The population surveyed considered the implementation of a local rice husk processing plant to be a good project idea that would generate employment and higher income for Tolima. 96.70% expressed their interest in participating in the implementation of this project. The demographic study indicates good acceptance by farmers, primary and secondary producers, companies, and citizens, meaning there is social potential for a rice husk processing plant. The perception of the community regarding the rice husk processing plant was positive; survey participants considered that it would boost the economy of the region through different innovation processes, such as taking advantage of rice production residues. It should be noted that the community was willing to participate in the different phases of design, implementation, and operation of the plant, thus creating different sources of employment for the area.

The recovery of the new raw material must be considered, since rice husks are currently not widely used by companies or small producers. Therefore, there are great opportunities for reuse of this industrial waste and furthermore, the implementation of this technology should reduce expenses caused by the disposal of the waste, increasing profits through savings.

## Discussion

### Financial viability

An economic analysis was performed to evaluate the financial viability of using rice husks to create biooil, biogas, and biochar through pyrolysis. The NPV mathematical model^38^ yielded a value of $17,765.73 USD, which is greater than zero, and the financial model predicted that the internal rate of return (IRR) would be 26%, which was greater than the opportunity interest rate (OIR) of 20 percent^39^. Using these terms, the predicted income from sales of the three biofuels was $210,684.54, constant over the five years of the planning horizon. Production costs, administration expenses, and financial expenses for years 1, 2, 3, 4 and 5 were $165,546.08, $172,595.04, $180,056.89, $188,014.13, and $196,504.52, respectively. Likewise, the earnings and/or profits at the end of each year were predicted to be $45,138.47, $38,089.50, $30,627.65, $22,670.41, and $113,288.10, respectively. Tables 2 and 3 show the initial investments in equipment, furniture, and supplies for the chemical plant and the administrative area.

**Table 2:** Initial investments in equipment, furniture, and supplies for the chemical plant.

Equipment and machinery	Unit #	Unit value	Cost (USD)
Silo	1	$5,684.40	$5,684.40
Conveyor belt	1	$4,111.68	$4,111.68
Rice husk dryer	1	$10,149.69	$10,149.69
Rice husk mill	1	$8,120.57	$8,120.57
Pyrolysis reactor	1	$81,205.68	$81,205.68
Cyclone	2	$1,979.39	$3,958.78
Capacitor	1	$12,849.00	$12,849.00
Filter	1	$3,654.26	$3,654.26
Storage tanks	4	$17,053.19	$68,212.77
Centrifugal pump	1	$269.32	$269.32
		Total	$19,8216.14

**Table 3:** Initial investments in equipment, furniture, and supplies for the administrative area.

	Unit value (USD)	Unit #	Cost (USD)
Furniture and equipment			
Computer equipment	$770.94	2	$1,541.88
Lighting	$5.88	10	$58.85
Office chairs	$51.37	2	$102.74
Office desks	$47.52	2	$95.03
Filing cabinets	$128.46	1	$128.46
Ambiance center	$308.12	1	$308.12
Supplies			
File folder	$7.68	10	$76.84
Box of spheres	$2.34	10	$23.39
Highlighter box	$4.09	10	$40.86
Notepad	$1.08	20	$21.59
Tape	$4.60	5	$23.00
Stapler	$3.06	2	$6.12
Drilling machine	$3.74	2	$7.48
Sewing hook box	$1.25	5	$6.27
AZ	$2.00	10	$20.04
Bins	$16.68	5	$83.39
Buckets 12 L	$3.32	5	$16.58
Broom	$2.80	3	$8.40
Mop	$3.08	3	$9.24
Dustpan	$5.11	3	$15.34
		Total (USD)	$2,593.61

The total initial investment was no more than the sum of the totals in Tables 2 and 3, or $200,809.75. Operational costs, such as costs of raw materials, labor, leases, and staffing, were also taken into account. The raw material costs were calculated using the amount of rice husk required, which is 157,943 kg/year, and the unit cost of the raw material, which is $0.31/kg of rice husk. An increase in the unit price of the raw material was considered for the following four years according to the inflation forecast in Colombia for the period 2022–2025. The cost of raw materials is presented in detail in Table 4.

**Table 4:** Raw materials cost.

Concept	Period					
	0	1	2	3	4	5
Quantity of raw material (kg)	–	157,943	157,943	157,943	157,943	157,943
Raw material cost per kg	–	$0.31	$0.32	$0.33	$0.34	$0.35
Raw material costs	–	$48,705.83	$50,215.71	$51,722.18	$53,273.85	$54,872.06

The labor cost was the salary remuneration of workers permanently employed in the plant: the two operators and the supervisor or production manager. It was assumed that the basic salary of the supervisor was $770.94/month and that of the operators $385.47/month. For the monthly payroll consignment, the income of these employees was taken into account, which was the basic salary and transportation subsidy ($27.36 for the first year) for those who earn less than 2 SMMLV, meaning it only applied to operators, and the deductions of 4% of the basic salary for payment to the pension system and 4% for payment to the health system. Thus, the net monthly payable was the difference between the monthly income minus the monthly deductions and multiplication of by the 12 months of the year, which resulted in the total annual payroll for each employee. The sum of these values was $17,678.91. Table 5 shows the production costs to implement the process.

**Table 5:** Production costs.

	Period					
	0	1	2	3	4	5
Raw material costs	–	$48,414.75	$49,915.61	$51,413.07	$52,955.47	$54,544.13
Labor costs	–	$26,543.87	$28,017.12	$29,595.81	$31,288.50	$33,104.50
Staffing	–	$367.84	$379.24	$390.62	$402.34	$414.41
Leases (80%)	–	$24,522.64	$26,484.45	$28,603.21	$30,891.47	$33,362.79
Total (USD)	0	$99,849.10	$104,796.42	$110,002.72	$115,537.77	$121,425.82

The operating expenses refer to the salaries of the administrative personnel, which are the manager, secretary, and janitor, together with the remaining 20% of the rent. The basic salary of the manager was assumed at $1021.78/month, that of the secretary $383.17/month, and that of the janitor $232.08/month. With these values, the monthly and annual payroll of these workers, their social benefits and the contribution to social security and parafiscal systems were determined, as with the plant employees. The total income, which is no more than the sum of the income left by each of the products of the pyrolysis process when distributed and sold, amounted to $209,425.42. Table 6 presents income from the production of biochar, bio-oil, and biogas from rice husks.

**Table 6:** Income from production of biochar, bio-oil, and biogas.

Concept	Period					
	0	1	2	3	4	5
Biochar income	–	$42,867.22	$42,867.22	$42,867.22	$42,867.22	$42,867.22
Bio-oil income	–	$137,043.56	$137,043.56	$137,043.56	$137,043.56	$137,043.56
Biogas income	–	$29,514.64	$29,514.64	$29,514.64	$29,514.64	$29,514.64
Total income (USD)	–	$209,425.42	$209,425.42	$209,425.42	$209,425.42	$209,425.42

**Figure 5:**
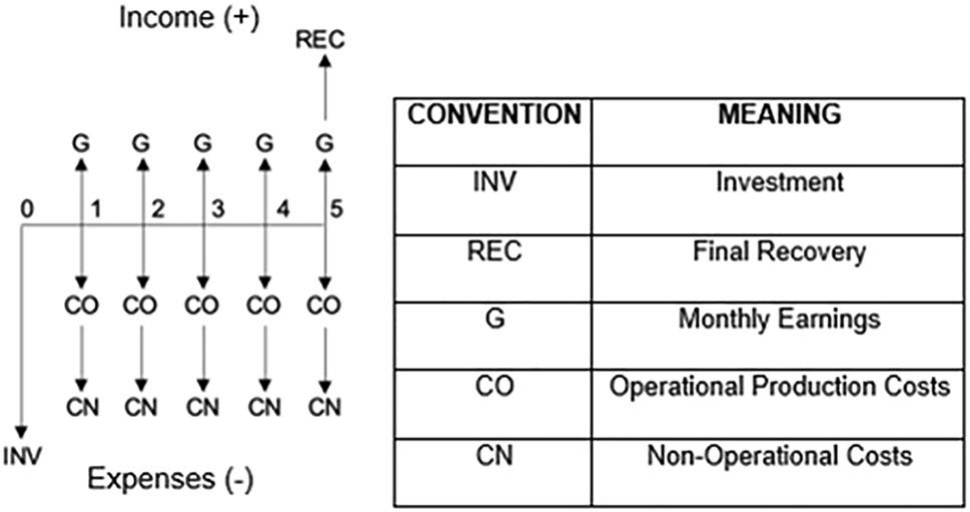
Triple bottom line diagram (TPL).

Once the income and expenses were calculated, the Net Present Value (NPV) and the Internal Rate of Return (IRR) mathematical models were implemented to determine whether the project could be viable or not, once the following assumptions are met: NPV > 0 and IRR > IOR. IOR represents the investor's opportunity interest; i.e., the profits that the investor expects from the investment in the project. For this reason, the cash flow was schematized with the monetary inflows and outflows of the project (Fig. 5).

The Net Present Value (NPV) indicator was positive ($17,659.56) or rather, greater than 0 and, similarly, the Internal Rate of Return (IRR) was greater than the Opportunity Interest Rate (OIR) at 6% and, therefore, the project was predicted to have economic viability and to have a return greater than that required by the investor. It is therefore calculated to be feasible to execute the rice husk energy conversion plant with the aspects taken into account in this study and that the profit expectations would be met, denoting an effective profitability of the project.

Finally, taking into account the above mentioned, a financial analysis was performed of the strategy to use rice husks to obtain biochar, bio-oil, and biogas by means of pyrolysis to thermally degrade this rice by-product in anaerobic conditions. Because the mathematical model NPV resulted in a value of $17,659.56, which is > 0, and the financial model IRR was 26%, which is > IOT = 20%, the strategy is thought to be profitable. Under these terms, the income due to the sales of the three biofuels was $209,425.42, constant over the 5-year planning horizon. The value of the salvage of the chemical plant equipment in the last year was $98,515.77; conversely, production costs, administrative expenses, and financial expenses for years 1, 2, 3, 4 and 5 were $164,556.72, $17,1563.56, $178,980.81, $186,890.49, and $195,330.14, respectively. Accordingly, the earnings and/or profits at the end of each year are Ps. $44,868.70, Ps. $37,861.86, Ps. $30,444.61, Ps. $22,534.93, and Ps. $112,611.05, respectively.

### Analysis of the triple bottom line (TPL)

**Figure 6:**
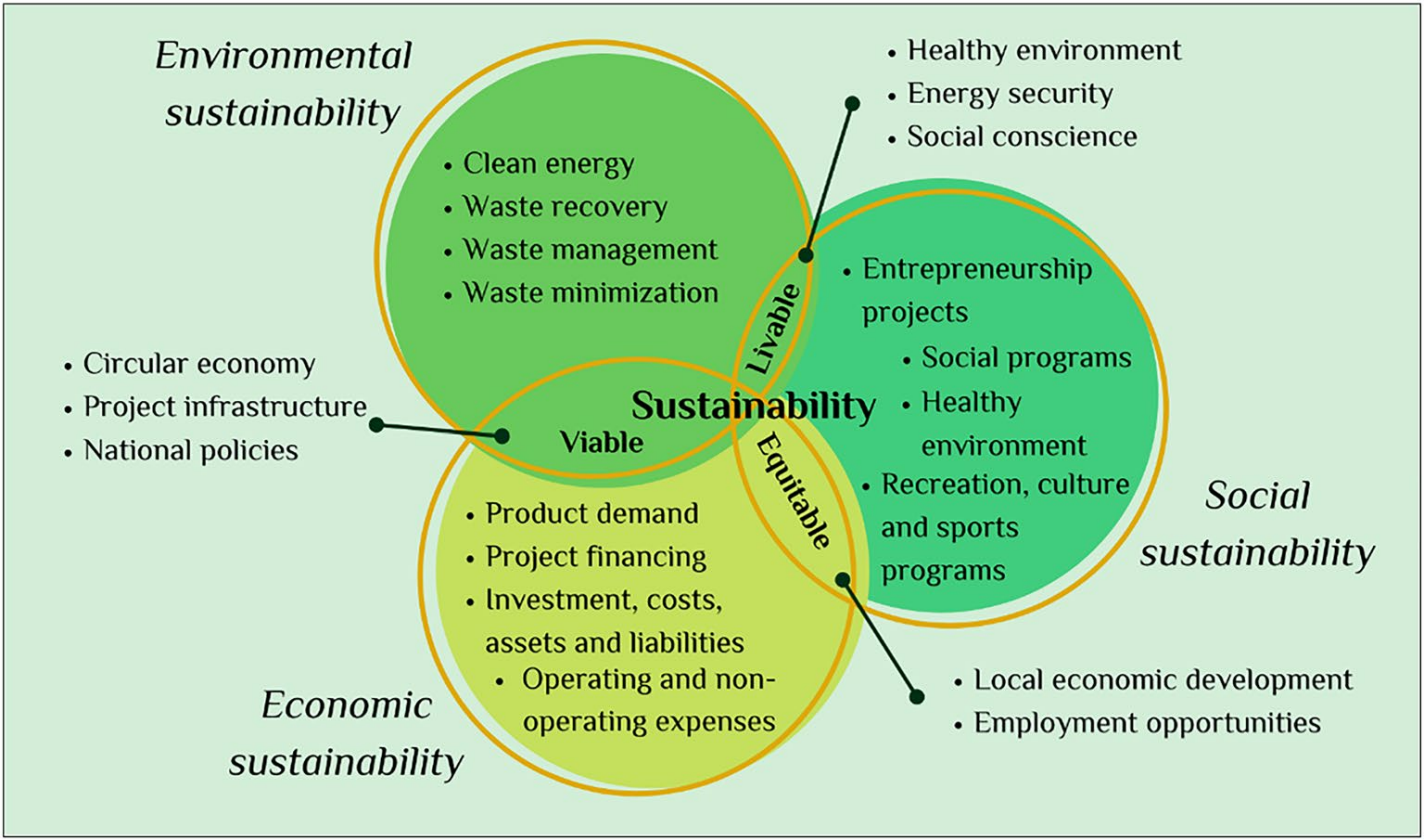
Triple bottom line diagram (TPL).

The following aspects were crucial for the analysis of the TPL and to form the triple account diagram of sustainability as shown in Fig. 6.

- Economic factor: The economic viability of this project was guaranteed for a period of 5 years. The project was predicted to be profitable because the NPV mathematical model had a value of $17,642.24, which was greater than zero, and the IRR was 26%, which was greater than the OIR (20%). The project could allow for economic development in Espinal, Tolima through the strengthening and implementation of new practices of use for rice husks and their inclusion as new secondary sector economic activities.
- Social factor: A potential positive impact emerged based on public perception of the plant and employment possibilities for people who can contribute their knowledge about the rice industry and who wish to learn about it. At the same time, greater job opportunities could be generated by requiring personnel to be continuously trained for the design, construction, and operation phases of the chemical plant and thereby promote social, cultural, recreational, and sports programs that ensure harmonious relations with the community and that allow people access to different activities and enjoy a healthy environment.
- Environmental factor: The rice husk processing plant allows rice husks to be used as a raw material to generate cleaner energy that reduces the carbon footprint by generating less pollution. This ensures the environmental viability of the project and the generation of more environmentally friendly products such as biogas, bio-oil, and biochar.

At present, there is a transcendence in the challenges that appear with the different problems at world level. Sustainable development is no longer only about focusing on processes that are environmentally sustainable but also must involve two very important factors that are interrelated to talk about true sustainable development: the economic factor and the social factor. This concept includes all three factors at the same time. If one factor is missing, the terms used are as follows: a project with social and economic factors is an equitable project; a project with economic and environmental factors is a viable project; and finally, a project with social and environmental factors is a livable project. A company, process, or project is sustainable when it has an explicit strategy of sustainability that continues to maximize profits and shareholder value, but, in turn, protects or rehabilitates the environment and contributes to improving society.

While countries such as the United States, Russia, and Saudi Arabia are still producing oil, the reality is that the future availability of oil is uncertain because it is a non-renewable resource. In addition, the oil industry has a significant environmental impact both upstream and downstream, including negative effects on soil, water, air, fauna, and flora. Although there is legislation on processes and techniques used in projects for improved hydrocarbon recovery, social programs, and an evaluation of environmental impacts, alternatives must be studied in order to use by-products from other industries to counteract these effects.

Therefore, techniques and processes have been rethought to manage the triple bottom line of sustainability, i.e., environmental, economic, and social sustainability, in accordance with the need of industries to increase productivity without leaving aside the reduction of the environmental and social footprint. Circular economy, orange economy, bioeconomy, implementation of the 7 R's, and circular production cycles are some terms or concepts commonly used. The use of biomass that conventionally represented a waste or residue to obtain value-added products is undergoing a boom, due to the potential to obtain energy from gasification, combustion, and/or pyrolysis processes. This has allowed for the generation of products such as biogas, bio-oil, and biochar as biofuels from resources that are generated every day from other economic activities and that do not raise environmental pollution rates, as is the case with fossil fuels.

## Conclusion

Rice husk is a by-product of the rice industry that represents an opportunity for the development of circular economy projects, one of which is a processing plant in Tolima, Colombia for rice husks as a sustainable energy alternative, a project which presents environmental, social, and economic viability.

Rice husks are rich in macro and micronutrients typical of this residue of vegetable origin, where the main macronutrient is cellulose biopolymer and the main micronutrient is silica (silicon dioxide). Rice husks have a relatively low humidity; their appearance is dry and does not allow the penetration of intermolecular spaces by water. Rice husks are characterized by being neutral and having a significant presence of organic carbon, which promotes a low decomposition rate and leads to a high potential for waste recovery. This study shows that there can be economic, social, and environmental sustainability to using rice husks as biomass in the chemical process of pyrolysis to obtain biochar, biogas, and bio-oil as bioproducts. This could be one of a myriad of solutions needed to address current problems associated with the energy crisis of the planet.

Additionally, the implementation of this energy sustainability strategy could lead to economic development in the community of Espinal, Tolima. This could be a supplementary economic activity to the production of rice and could generate an increase in the labor supply by requiring labor during the stages of production, design, construction, and operation of the processing plant. The efficiency of the reboiler and the heat transferred inside the combustion engine are constant over time; that is, their trend is linear without pronounced increases or decreases. This means that this equipment should work correctly for at least one year and should not have failures that reduce efficiency over time.

Sustainable energy is when energy efficiency is derived from renewable sources. This proposal supports a balance among energy security to ensure easy access to energy for consumers, competitiveness in economic terms, and environmental sustainability. There is a need to switch to alternative energy sources from biomass to replace fossil fuels that have generated a fairly pronounced social and environmental impact. Rice husks have the ideal physicochemical properties to be transformed into energy by processes such as pyrolysis, gasification, and combustion. Rice husks are not currently widely reused by companies and small producers, becoming an industrial waste with great possibilities of use. Due to their composition, they are easy to burn, being an ideal raw material for the clean generation of electric energy. This energy can be considered clean because it uses a residue that produces greenhouse gases with the purpose of eliminating them quickly producing energy, helping to create an energetic sustainability, which benefits the environment, helps companies to reduce costs and makes a social impact creating new sources of employment and the possibility of having energy in sectors where it cannot be easily supplied.

In accordance with the optimization of resources, in this case of rice husks as agricultural waste from the rice industry, the need for raw materials is reduced, and the implementation of waste is stimulated given its energetic properties. A circular economy model capable of ensuring sustainable and progressive growth in which the theory that everything has value is evident, since rice husks are being recycled and their useful life is extended as potential biomass in the conversion into biofuels.

## Data Availability

The datasets generated during and/or analyzed during the current study are available from the corresponding author on reasonable request.

## Abbreviations

SAM: System advisor model software
NPV: Mathematical model of net present value
IRR: Internal rate of return
OIR: Opportunity interest rate
FAO: Food and Agriculture Organization of the United Nations
LCA: Life cycle analysis
LCOE: Leveling cost of energy
TPL: Triple bottom line
PFD: Process flow diagram
